# Risk Stratification in Primary Biliary Cholangitis

**DOI:** 10.3390/jcm12175713

**Published:** 2023-09-01

**Authors:** Francesco Martini, Daniele Balducci, Martina Mancinelli, Valerio Buzzanca, Elena Fracchia, Giuseppe Tarantino, Antonio Benedetti, Marco Marzioni, Luca Maroni

**Affiliations:** Clinic of Gastroenterology, Hepatology, and Emergency Digestive Endoscopy, Università Politecnica delle Marche, 60126 Ancona, Italy; daniele.balducci01@gmail.com (D.B.); martinamanci@gmail.com (M.M.); valerio.buzzanca@gmail.com (V.B.); fracchiaelena@gmail.com (E.F.); giuseppe.tarantino@ospedaliriuniti.marche.it (G.T.); a.benedetti@univpm.it (A.B.); m.marzioni@staff.univpm.it (M.M.); l.maroni@staff.univpm.it (L.M.)

**Keywords:** primary biliary cholangitis, risk stratification, precision medicine

## Abstract

Primary Biliary Cholangitis (PBC) is a chronic cholestatic liver disease with a heterogeneous presentation, symptomatology, disease progression, and response to therapy. The current risk stratification assessment, aimed at identifying patients with a higher risk of disease progression, encompasses an in-depth analysis of demographic data, clinical and laboratory findings, antibody profiles, and the evaluation of liver fibrosis using both invasive and noninvasive techniques. Treatment response scores after one year of therapy remain to date a major factor influencing the prognosis of PBC patients. While the initial therapeutic approach with ursodeoxycholic acid (UDCA) is universally applied, new second-line treatment options have recently emerged, with many others under investigation. Consequently, the prevailing one-size-fits-all approach is poised to be supplanted by tailored strategies, ensuring high-risk patients receive the most appropriate treatment regimen from diagnosis. This will require the development of a risk prediction model to assess, at the time of diagnosis, the course, outcome, and response to first and additional treatments of PBC patients. This manuscript provides a comprehensive overview of the current and emerging tools used for risk stratification in PBC and speculates on how these developments might shape the disease landscape in the near future.

## 1. Introduction

Primary Biliary Cholangitis (PBC) is a chronic cholestatic liver disease characterized by the destruction of small- to medium-sized intrahepatic bile ducts, leading to cholestasis, fibrosis, and potentially cirrhosis and liver failure [1]. This condition predominantly affects middle-aged women, with a female-to-male ratio of approximately 9:1. Despite its relative rarity, PBC is a significant cause of morbidity and mortality among liver diseases. The pathogenesis of PBC is complex and multifactorial, involving genetic predisposition, environmental factors, and immune-mediated processes. The hallmark of PBC is the presence of anti-mitochondrial antibodies (AMA) in the serum of 90–95% of patients [2,3], targeting the E2 subunit of the pyruvate dehydrogenase complex (PDC-E2). Due to its high specificity, a positive AMA serology in patients with chronic cholestasis can confidently establish the diagnosis of PBC without requiring a liver biopsy [4,5]. The immune response against PDC-E2-expressing biliary epithelial cells leads to chronic inflammation and the progressive destruction of the bile ducts. Clinically, PBC presents with a wide spectrum of manifestations, ranging from asymptomatic disease detected incidentally due to abnormal liver function tests (LFTs) to symptomatic disease with pruritus, fatigue, and complications of cirrhosis. The course of this condition is highly variable, with some patients remaining stable for many years, while others progressing rapidly to advanced stages. A considerable proportion of patients advance to cirrhosis and its complications, including an increased risk of hepatocellular carcinoma (HCC). While there has been a relative decrease over the past few decades, the absolute rates of liver transplantation (LT) for PBC in Europe remain steady [6], representing one of the common indications for LT. Treatment strategies for PBC have evolved significantly in the last few years. Ursodeoxycholic acid (UDCA) has been the mainstay of treatment, improving biochemical parameters and slowing disease progression. However, approximately one-third of patients do not respond adequately to UDCA. For these patients, second-line therapies such as obeticholic acid (OCA) have been recently approved [7], and several other drugs are currently in clinical trials.

The risk assessment of PBC patients can significantly differ, influenced by a multitude of factors such as their age, gender, autoantibody profile, biochemical panel results at the time of diagnosis and during the treatment, and disease stage. Each of these elements plays a critical role in shaping an individual’s risk profile and can be instrumental in guiding treatment decisions. Risk stratification should be performed at the time of diagnosis and re-evaluated during therapy as it allows us to predict the hazard of disease progression and the rate of response to treatment. These concepts have been expanding in recent years because, having new therapeutic possibilities available, they allow us to implement precision medicine for patients. In this manuscript, we review the tools currently available for risk stratification in PBC and explore potential avenues for advancing beyond the current stratified medicine toward a more tailored and individualized approach.

## 2. Risk Stratification

The course of PBC can vary significantly among patients. Some may experience a slow progression of the disease, while others may develop advanced fibrosis and liver cirrhosis within a few years. To accurately assess each patient’s prognosis, healthcare providers must evaluate a variety of factors. These include demographic, clinical, laboratory, and serological parameters. Additionally, the patient’s response to treatment and the stage of the disease, determined by the extent of fibrosis, are also evaluated (Table 1).

### 2.1. Individual Factors

#### 2.1.1. Age

Demographic factors, including gender and age, should be taken into account when initially assessing a patient’s risk profile. Elderly patients (age at diagnosis > 55 years) have a standardized mortality rate similar to that of the general population, whereas younger patients have a mortality rate seven times higher than expected due to liver-related causes [8]. Age at diagnosis is strongly associated with the response to UDCA therapy and transplant-free survival; response rates range from 90% among patients older than age 70 to less than 50% for those younger than age 30 [9]. Specifically, younger age is linked to an increased risk of treatment failure, LT, and death, whereas the highest chances of responding well to UDCA therapy are observed in patients over the age of 65 [9,10].

#### 2.1.2. Sex

Although PBC predominantly affects females, there are notable differences between genders in terms of disease presentation and progression. Several studies have indicated that males tend to experience delayed diagnosis [9,11,12,13,14,15,16], resulting in older age at PBC identification and more advanced liver disease/cirrhosis [12,13,17,18,19,20]. Consequently, males have a poorer prognosis compared to females, with an elevated risk of developing jaundice, acute liver failure, spontaneous bacterial peritonitis, LT, and liver-related mortality [10,13,18,19,20,21,22,23].

Furthermore, there is evidence suggesting a disparity in the risk of HCC between male and female PBC patients. A meta-analysis conducted by Natarajan et al., which evaluated 18 studies, demonstrated that male gender is a risk factor for developing HCC, with an incidence rate of 9.82 per 1000 person-years compared to 3.82 per 1000 person-years in females [24]. Additionally, HCC incidence is higher in male UDCA nonresponders compared to responders, with a hazard ratio of 4.44 [25]. Harada et al. proposed that the lower occurrence of HCC in female PBC patients could be attributed to estrogen-mediated preventive mechanisms. In females, the risk of HCC increases proportionally with the histological stage of liver damage, while in males, HCC can occur at any stage [26].

Data on the gender-stratified response to UDCA treatment are, however, more conflicting. Some studies have shown that males tend to have a lower response rate to UDCA therapy [9,10,27,28], while others have demonstrated that gender has no significant impact on treatment response [19,21,29,30,31,32,33]. These discrepancies may be attributed to factors such as small sample sizes in certain studies, retrospective study designs, and, importantly, variations in the criteria used to define treatment response [34]. A study evaluated the role of gender in the response to OCA therapy and found no significant differences between males and females [35].

### 2.2. Clinical Factors

Most PBC patients are typically diagnosed when asymptomatic. When symptoms are present, the most commonly reported include fatigue, pruritus (itching), and sicca complex (dryness of the eyes and mouth). These symptoms adversely affect the quality of life [36,37,38], but there is conflicting evidence regarding their effect on the prognosis of PBC patients.

#### 2.2.1. Symptomatic Disease

Some studies suggest that patients with a symptomatic presentation of PBC may have a poorer response to UDCA therapy (63% vs. 81%) [39], an increased risk of developing cirrhosis and associated complications (31% vs. 13%), and worse survival compared to asymptomatic patients [40]. On the other hand, additional studies suggest that the absence of symptoms may simply indicate an earlier stage of the disease with better biochemical profiles [41]. Thus, the presence of symptoms may be more indicative of the disease stage rather than an independent factor affecting prognosis [42,43]. The potential additional prognostic value of symptoms in existing risk stratification models remains unknown and requires further investigation in prospective studies.

#### 2.2.2. Extrahepatic Autoimmune Diseases

PBC is frequently associated with other autoimmune conditions, collectively known as extrahepatic autoimmune diseases (EHAIDs). The prevalence of EHAIDs among PBC patients is estimated to be around 30%, with autoimmune thyroid diseases, Sjögren’s disease, systemic sclerosis, rheumatoid arthritis, systemic lupus erythematosus, celiac disease, psoriasis, and inflammatory bowel diseases (IBDs) being the most frequently observed. EHAIDs are more prevalent in women, except for IBDs, which are more common in men [44]. While there is often an overlap between PBC and EHAIDs, the presence of EHAIDs does not seem to significantly impact the clinical presentation or outcomes of PBC [45].

### 2.3. Variant Syndromes

Variant syndromes typically refer to conditions that share some clinical, biochemical, or histological features with PBC but also exhibit characteristics of other liver diseases. These syndromes have a different disease course, making early diagnosis crucial for the establishment of an appropriate treatment and management plan.

#### 2.3.1. AIH-PBC Overlap Syndrome

The term “overlap syndromes” is used to describe various conditions characterized by the clinical, biochemical, immunologic, histologic, or cholangiographic features shared by the most common autoimmune liver diseases: PBC, Autoimmune Hepatitis (AIH), and Primary Sclerosing Cholangitis (PSC) [46]. Among these, the AIH-PBC overlap syndrome (AIH-PBC OS) is the most studied and prevalent. It is found in approximately 8–10% of patients with PBC who present with histological, biochemical, and clinical characteristics resembling AIH. If patients with PBC do not respond to first-line UDCA therapy after 6-12 months or present with markedly elevated liver enzymes, AIH-PBC OS should be ruled out [5]. According to the European Association for the Study of the Liver (EASL) guidelines, the Paris criteria are commonly used to diagnose AIH-PBC OS [5,47]. These criteria include:

- two of the following: (A) alkaline phosphatase (ALP) > 2× upper limit of normal (ULN) or gamma-glutamyltransferase (GGT) > 5× ULN; (B) AMA > 1:40 or PBC-specific antinuclear antibodies (ANA) (immunofluorescent or/and specific anti-sp100/gp-210 test); (C) florid bile duct lesion on histology;
- and two of the following three features: (A) alanine aminotransferase (ALT) > 5× ULN; (B) Immunoglobulin G (IgG) serum levels > 2× ULN or smooth muscle antibody positive (SMA); (C) moderate or severe interface hepatitis on histology.

A liver biopsy is, however, considered mandatory for diagnosing AIH-PBC OS as it provides information about the features of liver injury (interface hepatitis and fibrosis), while also helping to rule out other possible diagnoses [5]. The detection of this condition is crucial due to its poorer long-term prognosis compared to “pure PBC” or AIH without overlap, as it demonstrates an accelerated progression of liver fibrosis (69.6% vs. 46.2%), liver-related death, and the need for LT [48,49].

UDCA monotherapy may induce a biochemical response in some patients with PBC-AIH OS, but most patients may require a combination of UDCA and immunosuppressive therapy to obtain a complete response [46]. In addition, the use of combination therapy in non-cirrhotic patients has been associated with reduced rates of fibrosis progression compared to those receiving UDCA monotherapy [50].

#### 2.3.2. Premature Ductopenic Variant

The premature ductopenic variant is a specific form of PBC characterized by a rapid and significant loss of bile ducts (more than 50% of the ductal network) [51]. This variant occurs in approximately 5–10% of PBC patients [52]. This condition should be suspected when severe itching and cholestatic jaundice are present, without signs of hepatocellular failure or portal hypertension. The severity of itching experienced by patients can greatly diminish their quality of life. To manage this intense discomfort, a variety of therapeutic strategies are often implemented, potentially leading to an early fulfillment of LT criteria. The chronic and severe cholestasis experienced in this variant can lead to nutritional deficiencies due to impaired absorption of fats [51]. Identifying significant ductopenia on a liver biopsy during the initial assessment can serve as an important predictor of a poor response to the standard UDCA therapy and histological progression [52] and may influence the evaluation of novel therapeutic approaches [53].

### 2.4. Antibody Profile

The serological profile of PBC patients can offer valuable information for both diagnosis and prognosis. One of the diagnostic steps for PBC is the detection of AMA and PBC-specific ANA, such as anti-gp210 and anti-sp100 [5].

#### 2.4.1. AMA

AMA, which target the PDC-E2, are found in over 90% of PBC patients, making them a highly sensitive indicator of the disease [54]; however, AMA reactivity alone is not sufficient for a definitive PBC diagnosis. Despite the high sensitivity of AMA in detecting PBC, neither the presence of AMA nor their titer has been found to hold any prognostic value [3,55,56,57]. Conversely, AMA-negative PBC might have poorer outcomes. Despite the limited number of studies and small sample sizes, it has been observed that AMA-negative patients have a significantly lower survival rate free from liver-related complications, including LT and death, compared to AMA-positive ones [58].

#### 2.4.2. ANA

ANA are found in approximately 30% of PBC. Although their presence is specific to PBC, they have relatively low sensitivity. Certain immunofluorescent staining patterns, such as nuclear dots and perinuclear rims, suggest anti-sp100 and anti-gp210 reactivity, respectively. These patterns are particularly useful in diagnosing PBC in the small percentage of patients who test negative for AMA [59,60,61]. PBC-specific ANA appears to be linked to different patterns of disease progression, offering potential insights into the prognosis of the disease [62].

Anti-gp210 antibodies have been found to be associated with a more aggressive form of the disease, indicating their potential prognostic value [63]. This aspect has been confirmed in various studies over the years. A comprehensive meta-analysis led by Huang et al., which included five retrospective studies and a total of 737 East Asian patients, thoroughly evaluated the prognostic value of Gp210 antibodies at the time of diagnosis. The findings revealed a higher incidence of liver failure (RR = 5.77, 95% CI: 2.9–11.48) and mortality (RR = 2.38, 95% CI: 1.62–3.51) among patients who tested positive for anti-gp210 antibodies [64]. In 2020, Haldar et al. examined a single-center cohort of 499 PBC patients from the UK. Their findings revealed that patients with anti-gp210 antibodies had higher baseline levels of transaminases and bilirubin, as well as liver stiffness measurements exceeding 9.6 kPa at diagnosis; additionally, these patients had an increased risk of all-cause mortality or LT (HR = 3.22, 95% CI: 1.49–6.96) [65]. They were also less likely to respond to UDCA treatment (16.7% vs. 39.3%) and, interestingly, the five-year transplant-free survival rate was lower compared to those anti-gp210 negative (75.7% vs. 90.4%) [65]. The prognostic role of anti-gp210 antibodies has been recently confirmed in a large cohort study [66]. Therefore, the inclusion of anti-gp210 antibodies into the UK-PBC and GLOBE scoring systems could potentially enhance their risk stratification capabilities [67].

Anti-sp100 antibodies have been extensively studied, but their clinical significance in PBC remains a topic of ongoing discussion. A small-scale study conducted by Gatselis et al. demonstrated that a decrease in anti-sp100 titers, rather than anti-gp210, was linked to a more favorable response to UDCA treatment and, importantly, an improvement in the Mayo risk score [68]. In line with this, an increase in antibody titers has been associated with a poorer outcome [69]. Interestingly, another study found an inverse relationship between anti-sp100 antibody titers and the degree of liver fibrosis [70], adding another layer of complexity to our understanding. Despite these seemingly contradictory findings, it can be deduced that fluctuations in autoantibody levels, whether they increase or decrease, could signify a physiological shift indicative of disease progression [70].

#### 2.4.3. ACA

ACA, typically associated with systemic sclerosis, have also been detected in PBC patients who do not have concurrent systemic sclerosis. In fact, ACA positivity is observed in approximately 9–30% of PBC patients [71]. Although the diagnostic and predictive values of ACA are unclear, studies conducted on Asian populations have indicated a significant correlation between the presence of ACA and the development of portal hypertension, even in the absence of synthetic liver failure [72,73]. The precise mechanism underlying this correlation is yet to be fully understood.

#### 2.4.4. Novel Autoantibodies

In 2015, two novel biomarkers were identified: anti-KLHL12, which targets the Kelch-like 12 protein involved in collagen export and ubiquitination, and anti-HK1, which targets hexokinase-1, an enzyme located in the outer mitochondrial membrane that plays a role in glucose metabolism and apoptosis [74,75,76]. Both of these biomarkers are highly specific for PBC (≥95%) and have demonstrated higher sensitivity than anti-gp210 and anti-sp100 antibodies. When combined with existing markers, they can enhance the diagnostic sensitivity for PBC [74]. Furthermore, these biomarkers have been associated with prognostic implications. Anti-HK1 seropositivity is linked to a higher likelihood of liver decompensation events and lower transplant-free survival [69]. Meanwhile, a study found an association between the presence of anti-KLHL12 antibodies with increased liver fibrosis and elevated bilirubin [77]. However, these findings need to be confirmed through further studies involving larger and more heterogeneous cohorts.

### 2.5. Disease Staging

#### 2.5.1. Histological Features

While liver biopsy is infrequently required for the diagnosis of PBC and is limited to atypical disease presentations, such as seronegative disease and overlap syndromes, it remains a useful tool for disease staging and prognosis [5]. It is now widely acknowledged that global risk assessment goes beyond biochemical response and requires comprehensive outcome stratification through fibrosis staging [78].

The florid duct lesion is considered the typical histological lesion in PBC but is observed in only 10% of liver biopsy specimens [5]. It is characterized by intense chronic inflammatory infiltration between cholangiocytes, eventually leading to the destruction of small interlobular bile duct cells and causing cholestasis [79]. Bile duct damage is accompanied by portal tract inflammation [80], often associated with mild interface hepatitis. The presence of severe interface hepatitis suggests a diagnosis of AIH or PBC-AIH OS [5]. Persistent ductal and periportal inflammation, along with bile duct damage, trigger periportal and portal collagen deposition, ultimately leading to the development of cirrhosis [81].

Classical histological staging systems for PBC include Ludwig and Scheuer’s systems, which combine inflammation, cholestasis, and fibrosis [82,83]. Although these staging systems have been widely used, they have some important limitations. The inter-observer and intra-observer reproducibility of these systems is not well-established [82,83]. Additionally, considering that fibrosis and inflammation can occur at different time points, exhibit different therapeutic responses, and have different prognostic significance, it is now generally accepted that evaluating them separately is crucial [84]. Harada et al. proposed a novel approach to histological disease staging (Nakanuma staging system), introducing the concept of PBC grading, which evaluates cholangitis activity and hepatitis activity, and determines the stage of disease based on the presence of fibrosis, orcein-positive granule deposition, and bile duct loss [85]. For disease staging, two out of the three criteria are sufficient for a correct evaluation [85]. This system has demonstrated superior prognostic value and better prediction of cirrhosis development compared to the classical systems [86]. However, it is limited by fair inter-observer reproducibility for staging evaluation and slight agreement for necroinflammatory activity [87]. Another scoring system proposed by Wendum et al. assesses fibrosis, ductopenia, and interface hepatitis separately and has shown a good correlation with biochemistry and better inter-observer agreement. Nevertheless, due to the short follow-up time, it lacks prognostic value [88].

#### 2.5.2. Noninvasive Markers of Fibrosis—Biomarkers

As liver fibrosis progression has been shown to predict survival in patients with PBC, repeated assessment of the fibrosis stage is crucial [89]. Notwithstanding, liver biopsy, which is still considered the gold standard for fibrosis staging in chronic liver diseases, is limited by possible side effects, sampling errors, and costs, making noninvasive markers of liver fibrosis desirable [90,91,92]. While the majority of serum biomarkers have been studied in patients with Hepatitis-B (HBV), Hepatitis-C (HCV), alcoholic fatty liver disease (ALD), and non-alcoholic fatty liver disease (NAFLD), only a few have been validated for PBC. 

The AST-to-platelet ratio index (APRI), initially developed for patients with HCV, has shown some value in predicting risk stratification in PBC [93]. APRI at diagnosis is associated with LT/death (HR = 1.95, 95% CI: 1.50–2.54); in particular, an APRI cut-off > 0.4 at baseline is predictive of LT/death (HR = 2.40, 95% CI: 1.32–4.36) and retain statistical significance at 1-year (HR = 2.75, 95% CI: 1.49–5.08), independently and additively of UDCA-response [93].

The Enhanced liver fibrosis (ELF) score, which combines metalloproteinase 1 (TIMP-1), serum hyaluronic acid (HA), and procollagen III N- terminal peptide (PIIINP), has been the only serological surrogate of liver fibrosis that has demonstrated good performance in detecting fibrosis staging according to two different histological classifications and identifying patients with poor prognosis; an ELF score of ≥10.0 predicts a higher incidence of clinical complications and worse survival [94].

Wisteria floribunda agglutinin (WFA)-positive Mac-2bp is another promising simple, noninvasive serological marker independently associated with liver fibrosis in patients with PBC, as verified by multiple studies [95,96]. 

#### 2.5.3. Noninvasive Markers of Fibrosis—Liver Stiffness

In addition to serum biomarkers, instrumental examinations can be used for fibrosis assessment. Liver stiffness, shown to be a good surrogate for liver fibrosis, can predict clinical outcomes associated with PBC [97]. 

Transient elastography (TE), the first ultrasound-based technique developed for noninvasive assessment of liver stiffness, utilizes mechanical impulses that generate shear waves in the hepatic tissue, which are then captured by an ultrasound transducer [98]. The main limiting factor of this method is a body mass index (BMI) > 28 kg/m [99], which can be partially mitigated with an XL probe [100]. Liver stiffness by TE is a well-established surrogate marker of fibrosis, and it is recommended by EASL for disease staging at baseline and during follow-up [98]. TE exhibits excellent diagnostic accuracy for cirrhosis (specificity > 90%) and good accuracy for fibrosis (specificity > 80%) [101]; it is easy to perform and provides instant results [102]. Corpechot et al. showed that a liver stiffness greater than 9.6 kPa at baseline (95% CI: 9.0–18.0) or an increase > 2.1 kPa/year (95% CI: 1.8–3.5) are good cut-offs for predicting the risk of future liver decompensation or LT [103]. Cristoferi et al. have identified that the best values to exclude or confirm advanced fibrosis are, respectively, liver stiffness by TE lower than 6.5 kPa (sensitivity = 0.91, negative predictive value = 0.96) and higher than 11.0 kPa (specificity = 0.99, positive predictive value = 0.94) [104]. A recent retrospective follow-up study, including almost 4000 patients with PBC, confirmed that elevated liver stiffness assessed by TE is independently associated with poor clinical outcomes (HR per kPa added = 1.065, 95% CI: 1.057–1.074) and improves the predictive value of biochemical response criteria, fibrosis scores, and prognostic scores [105]. Furthermore, liver stiffness stratifies patients in low- (<8 kPa), medium-, and high- (>15 kPa) risk groups [105].

More recently, a new method called two-dimensional shear wave elastography (2D-SWE) has been introduced, allowing real-time acquisition of shear wave propagation in liver tissue under B-mode ultrasound imaging [106]. This method has demonstrated a good correlation with histologic liver fibrosis in mixed cohorts of patients with chronic liver diseases, and although specific data on PBC patients are sparse, the preliminary evidence is promising [107,108].

Acoustic radiation force impulse (ARFI) is another ultrasound-based method that measures the speed of shear waves caused by acoustic pulses generated by local displacement of liver tissue [109]. Although extensively validated for viral hepatitis and NAFLD, caution should be exercised when interpreting shear wave speed in early-stage fibrosis in patients with PBC [110]. 

Besides ultrasound-based techniques, magnetic resonance (MR) imaging is another interesting method for noninvasive hepatic fibrosis assessment. The deep tissue penetration and absence of ionizing radiation allow repeated evaluations without safety concerns. MR elastography (MRE), similar to ultrasound-based elastography techniques, measures liver stiffness by analyzing mechanical waves propagating through the organ generated by an external driver. Compared to TE, MRE can characterize larger portions of liver parenchyma, is not limited by obesity [111], and has demonstrated superior accuracy in many chronic liver diseases [112,113]. A recent study by Osman et al. assessed the accuracy of MRE in PBC patients, finding that MRE can accurately detect advanced fibrosis with a cut-off of 4.60 kPa (AUC = 0.82), outperforming biochemical tests, and can predict hepatic decompensation and liver-related complication (HR = 2.09, 95% CI: 1.57–2.78) [97]. However, it showed inferior performance in differentiating early fibrosis stages compared to TE. Given the longer time of acquisition and the elevated cost, it is reasonable to reserve this technique for patients with high BMI and those who need cross-sectional imaging [97].

Both histological evaluation and noninvasive markers of fibrosis provide valuable tools for disease staging in PBC, facilitating prognosis assessment and guiding treatment decisions. It is important to note that while biochemical markers and established elastography methods have been extensively validated in large clinical trials, imaging methodologies have shown promise in assessing fibrosis in PBC but require further research and validation to establish their broader utility and overcome limitations, especially in early-stage fibrosis stages. Continued research in this field is crucial to enhance diagnostic accuracy, improve prognostic prediction, and optimize patient care.

### 2.6. Liver Biochemistry

#### 2.6.1. ALP

The relationship between serum ALP and the risk of LT and death in patients with PBC has been found to be log-linear, with higher levels of ALP indicating a decreased chance of transplant-free survival. A meta-analysis of almost 5000 PBC patients revealed that an ALP level > 2× ULN at baseline (HR = 2.13, 95% CI: 1.72–2.65) and after one year of follow-up (HR = 2.49, 95% CI: 2.14–2.89) had the highest predictive ability [114]. Therefore, ALP level is a reliable marker of treatment response, with lower levels indicating a better prognosis, decreased mortality, and longer transplant-free survival [115]. The prognostic potential of ALP is further enhanced when it is combined with either bilirubin [114] or GGT [115] levels in the serum. Despite the fact that GGT demonstrated a correlation with nonresponse to UDCA [33] and an increased risk of liver-related death and liver transplantation [116], it should not be used as the sole marker in evaluating the biochemical response to therapy [117].

#### 2.6.2. Bilirubin

The level of serum bilirubin plays a significant role in forecasting patient survival [118]. However, its effectiveness in stratifying risk at the onset of the disease is limited, as elevated levels are typically observed in the later stages of PBC [119]. Recent research suggests that total bilirubin possesses a varying ability to predict prognosis, even when its levels are within the normal range; specifically, values > 0.6× ULN are associated with worse outcomes [120].

### 2.7. Assessment of Response to Treatment

The advent of UDCA has had a profound effect on the natural progression of PBC, as it has been found to improve LFTs, liver histology, and prolong liver transplant-free survival [59]. Furthermore, a lack of improvement in LFTs despite UDCA therapy is associated with a poorer prognosis [27,121]. Consequently, a variety of prognostic models evaluating UDCA response have been devised to categorize patients based on their risk of developing liver failure. These tools can be split into two categories: those that yield qualitative outcomes based on binary variables (dichotomous scoring system), and those that evaluate the risk of the outcome over time using continuous parameters (continuous scoring system).

#### 2.7.1. Dichotomous Scoring System

Several approaches have been proposed to define the biochemical response to UDCA, with most of them being established retrospectively from small-to-medium-sized single-center longitudinal cohorts. These models aim to predict severe clinical outcomes, such as death or LT, and most have been validated at 12 months from the start of UDCA treatment. However, Zhang et al. suggest that a 6-month evaluation may have similar predictive performance [122]. Similarly, Murillo et al. identified a predictive threshold of nonresponse of ALP 1.9× ULN, at six months of UDCA therapy, that identifies eligible patients for early initiation of second-line treatment [123]. Dichotomous scores [52,115,121,124,125,126], which use different thresholds of bilirubin, transaminases, and ALP values, are used to categorize patients into responders and nonresponders. Responders are considered at low risk of progression, while nonresponders are at high risk. The best-known dichotomous models, as defined in Table 2, are variably accurate in predicting death or LT. The Paris I model is the most accurate, having a c-statistic of approximately 0,8. However, its sensitivity is low: 36% in the UK-PBC research cohort (N = 3165) [53]. Combining the Paris-I criteria with APRI after 1 year of UDCA treatment has been shown to improve risk stratification [93].

These scores are simple to use but have limitations in accurately predicting the risk of death from liver-related causes or the need for LT, as they fail to account for individuals with intermediate risk. Additionally, they do not indicate the time frame within which high-risk patients may reach the outcome [29].

#### 2.7.2. Continuous Scoring System

To address the limitations of dichotomous scores, the Global PBC Study Group and the UK-PBC consortium have developed continuous scoring systems, namely the GLOBE score [29] and the UK-PBC score [127] (Table 3). These scores incorporate both measures of treatment response at 12 months and parameters of disease severity. The GLOBE score takes into account the patient’s age, while the UK-PBC score includes the platelet count at diagnosis. In both risk scores, all predictor variables are continuous and treated as such. The UK-PBC risk score estimates the risk of liver transplantation or liver-related death occurring within 5, 10, or 15 years, while the GLOBE score predicts liver transplantation-free survival at 3, 5, 10, and 15 years.

The GLOBE and UK-PBC scores have demonstrated superior performance in predicting death or liver transplantation compared to the Paris-I criteria, with c-statistics at 15 years in the validation cohorts of 0.82 and 0.90, respectively [66]. The risk score calculators are accessible online at the respective websites (http://www.uk-pbc.com/resources/tools/riskcalculator/ (accessed on 10 July 2023), and http://www.globalpbc.com/globe (accessed on 10 July 2023)).

#### 2.7.3. UDCA Predictive Score—UDCA Response Score (URS)

The GLOBE and UK-PBC scores are widely recognized as effective tools for predicting patient prognosis, but they do have some limitations. One major issue is that there is currently no established threshold for distinguishing high-risk from low-risk patients, which means that these scores cannot be used to guide treatment escalation or de-escalation. Additionally, there is no confirmed data on how well these scores predict prognosis for patients on second-line therapy [128]. Another limitation is that these scores are unable to determine which patients are less likely to respond to UDCA before the commencement of treatment. To address this problem, the UK-PBC Research Group and Italian PBC Study Group have developed the UDCA Response Score (URS), which uses various pretreatment clinical and serologic variables to identify patients who are at high risk of failing UDCA monotherapy [129,130]. Although the URS has been externally validated and shown to be highly accurate, it has not yet been integrated into standard clinical practice. The score can be accessed online at the following website: https://www.mat.uniroma2.it/~alenardi/URS.html (accessed on 10 July 2023).

## 3. Risk-Stratified Management

**Figure 1:**
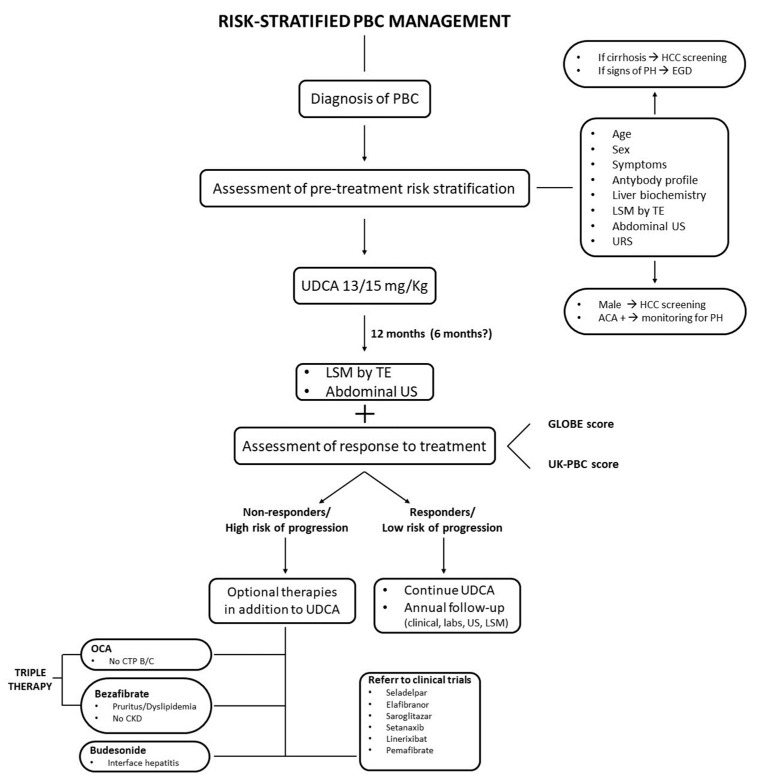
Risk-stratified management. Abbreviations: ACA: anti-centromere antibodies; CKD: chronic kidney disease; CTP: Child–Turcotte–Pugh; EGD: esophagogastroduodenoscopy; HCC: hepatocellular carcinoma; LSM: liver stiffness measurement; OCA: obeticholic acid; PBC: Primary Biliary Cholangitis; PH: portal hypertension; TE: transient elastography; UDCA: ursodeoxycholic acid; US: ultrasound.

Figure 1 outlines a proposed management strategy for patients diagnosed with PBC, based on the most recent and relevant data.

The process of risk assessment is initiated at the point of diagnosis. This involves a comprehensive evaluation that includes demographic factors (such as age and sex), laboratory findings (including LFTs), clinical and serological parameters (like the antibody profile), as well as an abdominal ultrasound and liver stiffness measurement using TE. If the initial evaluation reveals signs of liver cirrhosis or portal hypertension, the patient should, respectively, be enrolled in a screening program for HCC and undergo an endoscopy to exclude the presence of esophagogastric varices. Given their elevated risk, the American Association for the Study of Liver Diseases (AASLD) advocates for HCC surveillance in all male patients, despite the lack of cost-effectiveness analyses [4]. For individuals who are not part of an HCC surveillance program, it is suggested to regularly perform abdominal ultrasound screenings to check for portal hypertension. This is particularly important for those who have tested positive for ACA. The cause of portal hypertension in these individuals is not fully understood, and they may still develop the condition despite showing a good response to treatment and having normal LFTs. In the final step of pre-treatment risk analysis, the predictive value of response to UDCA therapy could be assessed with the URS score. This score, recently developed by Carbone et al. [129], has been shown to be accurate.

After 1 year of optimized therapy with 13–15 mg/kg/day of UDCA, a new risk re-stratification based on the biochemical response with continuous scoring systems (GLOBE and UK-PBC scores), should be performed. In nonresponder patients, who present a higher risk of disease progression, it is indicated to add a second-line therapy to UDCA, such as OCA. However, this is not recommended for patients with Child–Pugh B/C cirrhosis due to an increased risk of severe adverse events. Bezafibrate, although not currently included in existing guidelines, is often prescribed off-label in addition to UCDA/OCA and can be used as a first-line therapy for significant pruritus [131]. In nonresponders, it might be reasonable to perform a liver biopsy as it could provide additional information such as variant syndrome, moderate to severe interface hepatitis, steatosis, or steatohepatitis. For PBC patients exhibiting “florid” interface hepatitis on biopsy, budesonide has proven effective in improving the histological and biochemical stage when used in combination with UDCA [29].

## 4. Future Directions

Risk stratification modeling in PBC aims to identify patients who are at a higher risk of disease progression or nonresponse to treatment, allowing for personalized management approaches. 

Currently, patient risk stratification is primarily guided by therapeutic response scores. If a patient shows an inadequate therapeutic response after 12 months of UDCA therapy, OCA is added to the treatment. This represents the current sequential therapeutic approach applied worldwide. While this strategy has many benefits, such as a good therapeutic response in many patients and the safety and affordability of UDCA, it also has significant limitations. It may not promptly identify patients with poor response and disease progression (fibrosis and duct loss) or the onset of potentially irreversible symptoms.

The scientific community is therefore focusing on the possibility of adopting a different, top-down approach. This would allow for early and effective treatment in high-risk patients by combining two or more drugs simultaneously from the time of diagnosis.

In order to identify the ideal candidates for this new strategy, it is necessary to reshape current ideas about risk stratification in PBC. Indeed, the patient’s risk should be calculated at the time of diagnosis, while the therapeutic response scores at 12 months from the start of therapy would begin to lose importance. In contrast, there is a need for further studies on predicting treatment failure at the time of diagnosis [129]. This is in addition to ongoing studies that use high-throughput technologies to characterize an individual’s proteome, metabolome, microbiome, and epigenome, combined with clinical data, to provide comprehensive patient profiling [132,133,134,135,136,137,138]. In the future, Artificial Intelligence (AI) could revolutionize PBC management by enabling early diagnosis, predicting disease progression, and personalizing treatment strategies. AI algorithms can analyze complex datasets, potentially identifying novel biomarkers and therapeutic targets, thus paving the way for precision medicine in PBC [139,140,141].

In conclusion, the landscape of risk stratification in PBC is evolving. The traditional approach of sequential therapy based on therapeutic response scores at 12 months is giving way to a more proactive, top-down approach that considers the patient’s risk at the time of diagnosis. This shift is driven by the availability of new therapeutic options and the need to identify patients who could benefit most from these treatments. However, the new top-down strategy requires further refinement and validation. Future research should focus on developing predictive models for treatment failure at diagnosis and leveraging high-throughput technologies to provide a comprehensive profile of the patient. These advancements will enable a more personalized and effective management of PBC, ultimately improving patient outcomes.

## Figures and Tables

**Table 1:** Risk stratification in Primary Biliary Cholangitis (PBC) at baseline.

	Low Risk	High Risk
**Age**	>55 years	<55 years
**Sex**	Female	Male
**Clinical pattern**	No symptoms	Symptomatic disease AIH/PBC OS Premature Ductopenic Variant
**Antibody profile**	AMA-	Anti-gp210+ ACA+ Anti-HK1+ Anti-KLHL12+
**Biochemical panel**	Normal bilirubin ALP < 2× ULN	↑ bilirubin ALP ≥ 2× ULN APRI score > 0.54
**Histology**	No/Mild fibrosis	Advanced fibrosis/cirrhosis Interface hepatitis Ductopenia at the diagnosis
**Noninvasive markers of fibrosis**	LSM < 8 kPa/↑ < 2.1 kPa/y ELF score < 10.0 MRE < 4.6 kPa	LSM > 15 kPa/↑ > 2.1 kPa/y ELF score ≥ 10.0 MRE > 4.6 kPa

Abbreviations: ACA: Anti-centromere antibodies; AIH/PBC OS: autoimmune hepatitis/primary biliary overlap syndrome; AMA: anti-mitochondrial antibodies; APRI: AST-to-platelet ratio index; ELF score: enhanced liver fibrosis score; HK1: hexokinase-1; KLHL12: Kelch-like 12 protein; LSM: liver stiffness measurement; MRE: magnetic resonance elastography; ULN: upper limit of normal.

**Table 2:** Dichotomous scores of ursodeoxycholic acid (UDCA) response.

Score	Evaluation Time (mo)	Outcomes	Treatment Failure If	c-Statistics 5, 10, 15 Years
**Barcelona, 2006 [121]**	12	LTF survival	Decrease in ALP ≤ 40% and ALP ≥ 1× ULN	0.56, 0.61, 0.61
**Paris I, 2008 [115]**	12	LTF survival	ALP ≥ 3× ULN or AST ≥ 2× ULN or bilirubin > 1 mg/dL	0.81, 0.81, 0.80
**Rotterdam, 2009 [124]**	12	LTF survival	Bilirubin > 1 mg/dL and/or albumin < 1× ULN	NA
**Toronto, 2010 [52]**	24	LTF survival, histological progression	ALP ≥ 1.67× ULN	0.65, 0.70, 0.70
**Paris II, 2011 [126]**	12	LTF survival, ascites, variceal bleeding, encephalopathy	ALP ≥ 1.5× ULN or AST ≥ 1.5× ULN or bilirubin > 1 mg/dL	0.75, 0.75, 0.74
**Ehim, 2011 [125]**	6	LTF survival	Decrease in GGT ≤ 70% and GGT ≥ 1× ULN	NA

Abbreviations: ALP: alkaline phosphatase; AST: aspartate aminotransferase; GGT: gamma-glutamyltransferase; LTF: liver transplant-free; NA: not available; ULN: upper limit of normal.

**Table 3:** Continuous scores of ursodeoxycholic acid (UDCA) response.

Score	Evaluation Time (mo)	Outcomes	Variables	c-Statistics
**UK-PBC [127]**	12	Risk of LT or liver-related death within 5, 10, 15 years	At 12 months: ALP, AST, ALT, bilirubin At baseline: albumin, platelet	0.96, 0.95, 0.94 (at 5, 10, 15 years)
**GLOBE [29]**	12	LTF survival at 3, 5, 10 years	At 12 months: age, ALP, bilirubin, albumin, platelet	0.82

Abbreviations: ALP: alkaline phosphatase; AST: aspartate aminotransferase; ALT: alanine aminotransferase; LTF: liver transplant-free.

## Data Availability

Not applicable.
